# Increase in sickness absence due to mental disorders in Finland: trends by gender, age and diagnostic group in 2005–2019

**DOI:** 10.1177/1403494821993705

**Published:** 2021-02-20

**Authors:** Jenni Blomgren, Riku Perhoniemi

**Affiliations:** Research Unit, The Social Insurance Institution of Finland (Kela), Helsinki, Finland

**Keywords:** Sickness absence, mental health, mental disorders, depression, Finland

## Abstract

**Aims:**

Mental disorders are among the key public health challenges and cause a significant share of sickness absence. The aim of this study was to examine gender and age-specific trends in sickness absence in Finland among non-retired persons aged 16–67 years during 2005–2019 by main diagnostic groups. Special focus was put on the development of sickness absence due to mental and behavioural disorders.

**Methods:**

Data on compensated sickness allowance days were retrieved from the database of the Social Insurance Institution of Finland, and data on the non-retired population aged 16–67 years from the database of Statistics Finland for years 2005–2019. Yearly age-standardised sickness absence rates (yearly sickness absence days per each person in the population at risk) according to diagnostic group were calculated for women and men in age groups 16–34, 35–49 and 50–67 years.

**Results:**

A steep increase in sickness absence due to mental disorders was observed between 2016 and 2019 in all age groups among both genders, but the increase was more prominent among women. The age group 16–34 years also showed a longer-term gradual increase. In all examined gender and age groups, the increase was mainly a consequence of an increase in sickness absence due to depression and anxiety disorders.

**Conclusions:**

Increase in sickness absence due to mental disorders is an early sign of threats to work ability and productivity of the working-age population. Several factors may simultaneously drive the development. The specific reasons for the recent trend need to be studied.

## Introduction

Mental health problems are currently one of the key public health challenges of European societies [1]. Accordingly, increases in sickness absence due to mental and behavioural disorders have been reported especially during the 1990s and early 2000s [2–6]. Sickness absence entails significant losses of productivity and insurance costs in the working age population and is a key risk factor for disability retirement and permanent exclusion from the labour market [5, 7, 8].

In Finland, mental and behavioural disorders have long been the second largest cause of sickness allowance compensated by the Social Insurance Institution of Finland (Kela), after musculoskeletal diseases [9, 10]. After several years of only little change, a steep increase in sickness absence due to mental disorders has been observed after 2016. In terms of yearly compensated sickness absence days, mental disorders overtook the top position in 2018 [9, 10]. To understand this development better, an examination of trends in different population and diagnostic groups is needed.

In this study, we examine gender and age-specific trends in sickness absence in Finland among non-retired persons aged 16–67 years during 2005–2019 by main diagnostic groups, and with a special focus on the development of sickness absence due to mental and behavioural disorders.

## Methods

Sickness absence was measured through sickness allowance. Kela pays sickness allowance to non-retired persons aged 16–67 years as compensation for loss of income due to inability to work because of medically certified sickness or impairment. Sickness allowance can usually be paid starting from the 12th calendar day of sickness. Prior to the compensated period, the first days of sickness absence are normally covered by the employer through sick pay. However, sickness allowance can also be paid to those without employment. Data on compensated sickness allowance days were collected for years 2005–2019 from Kela’s database, including information on gender, age and diagnosis. Only sickness absences compensated by sickness allowance are included in the calculations, as there is no national register on shorter sickness absence spells.

Using the International Classification of Diseases, version 10 (ICD-10) [11], diagnoses were first categorised into the three most common diagnostic groups of sickness allowance in Finland: mental and behavioural disorders (ICD-10: F chapter; 34% of allowance days in 2019), musculoskeletal diseases (M chapter, 27%), and injuries (S and T chapters, 12%). All remaining causes, which were each clearly less common than the three above-mentioned groups, were classified into a fourth, residual group. Second, for a more detailed assessment, mental and behavioural disorders were further grouped into depression (F32–F33), other mood disorders (F30–F31, F34–F39), anxiety disorders (F40–F48) and other mental disorders (rest of the F chapter).

To construct the outcome measure, the sickness absence rate was calculated as the yearly total number of compensated sickness absence days per each insured person in the corresponding population group. In contrast to some other suggested measures of sickness absence [12, 13], the development of the yearly number of sickness absence days per the insured population depicts the total burden of sickness absence in the population during each year. As those retired are not eligible for sickness allowance, the non-retired persons aged 16–67 years at the beginning of each year; that is, those potentially at risk of sickness absence, constituted the insured population [13]. The numbers of population at risk were retrieved from Statistics Finland [14].

The total yearly numbers of persons in the population at risk varied between 3.15 and 3.22 million, and total yearly sickness allowance days were between 13.8 and 16.7 million during the period 2005–2019. Sickness allowance is paid for 6 days a week. The number of paid sickness allowance days was converted to true calendar time before calculating the sickness absence rate.

Age-standardised sickness absence rates were calculated using the pooled population of all years, women and men combined, as the standard population. Age standardisation was carried out using 5-year age categories. The differences between non-standardised and age-standardised figures were very small. The study required no ethical approval because data were collected from statistical databases.

## Results

**Figure 1.**
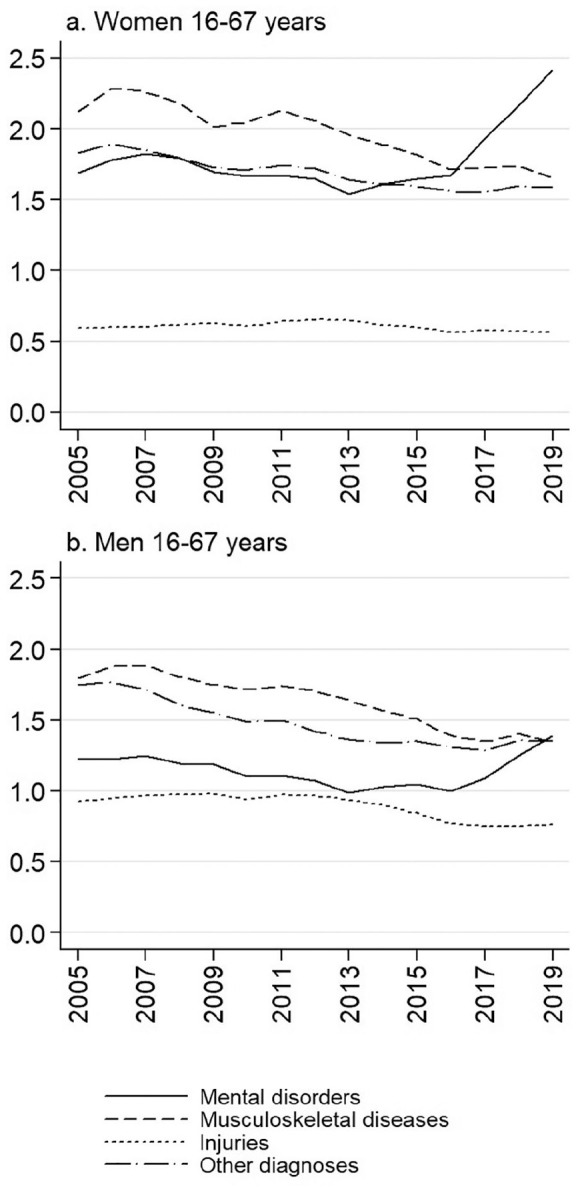
Age-standardised sickness absence rates due to major diagnostic groups in Finland, 2005–2019, for women and men. Yearly sickness absence days per each insured person.

Among both genders but more notably among women, there was a steep increase in sickness absence due to mental disorders between 2016 and 2019, while few simultaneous changes took place in other disease groups (Figure 1). Among women aged 16–67 years in 2019, the total adjusted sickness absence rate was 6.2, of which 2.4 days were caused by mental disorders. In men, the corresponding figures were 4.8 and 1.4. Sickness absence due to mental disorders topped musculoskeletal diseases in 2017 among women and in 2019 among men.

Among those aged 16–34 years, mental disorders have been the most important causes of sickness absence and increased during the whole observation period, while among those aged 35–49 years, mental disorders have been the most important cause since 2017. Among those aged 50–67 years, mental disorders are still far behind musculoskeletal diseases, but a recent increase has also been noted in this group (see Supplemental Figure 1).

**Figure 2.**
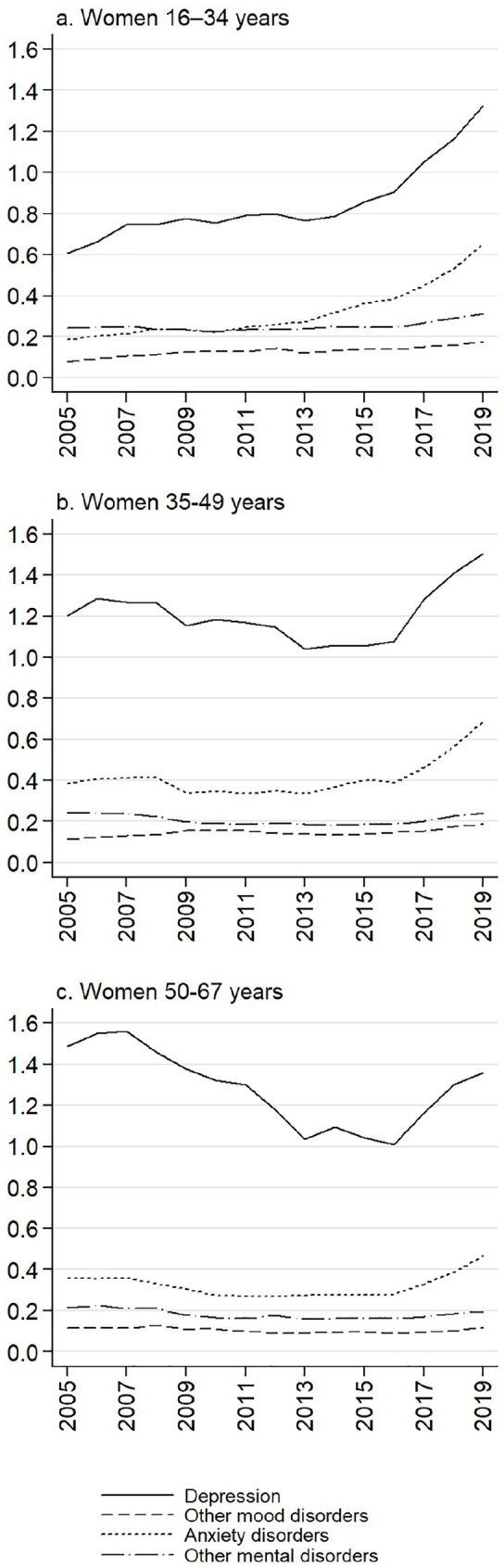
Age-standardised sickness absence rates due to mental and behavioural disorders by diagnostic group among women in Finland, 2005–2019, in three age groups. Yearly sickness absence days per each insured person.

**Figure 3.**
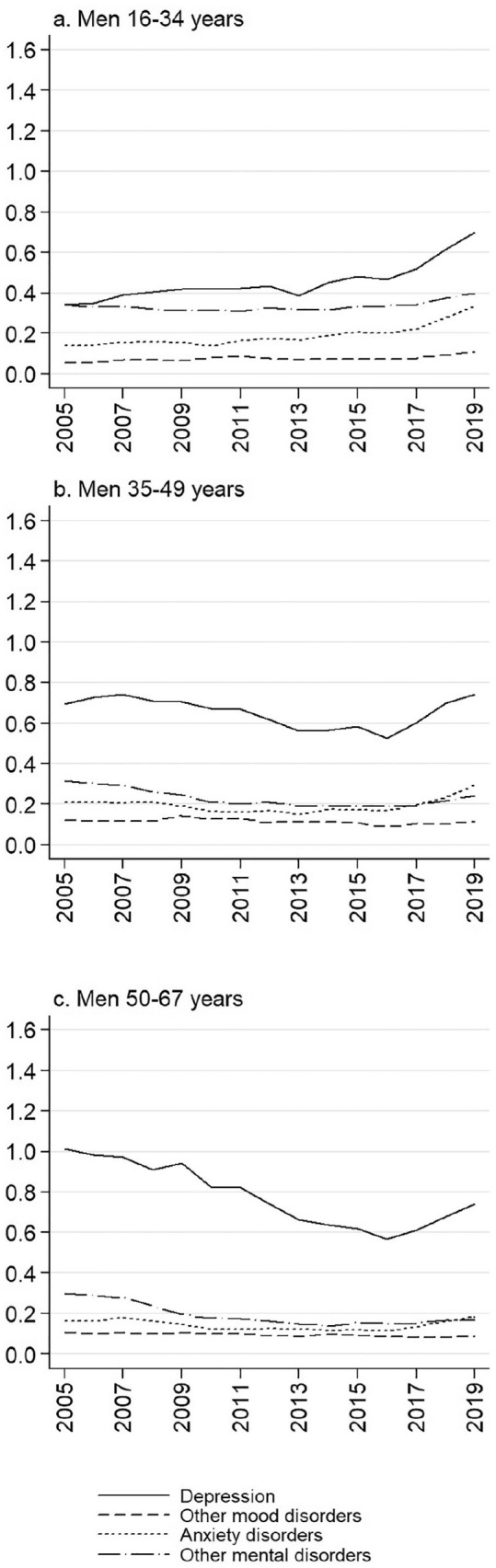
Age-standardised sickness absence rates due to mental and behavioural disorders by diagnostic group among men in Finland, 2005–2019, in three age groups. Yearly sickness absence days per each insured person.

Figures 2 and 3 show sickness absence rates by more specific diagnostic categories of mental disorders for women and men in three age groups. In all groups, depression is clearly the most important diagnosis causing sickness absence days. Among women, anxiety disorders are also prevalent. In all examined gender and age groups, the recent increase in sickness absence due to mental disorders is mainly attributable to an increase in depression and anxiety disorders, and the curves have been steeper than before after year 2016. While there has been a steady increase in the youngest age group, sickness absence due to depression and anxiety disorders has clearly also been increasing since 2017 in older age groups that have rather shown a decrease until year 2016.

## Conclusions

After a trend of overall decrease in sickness absence rates in Finland, there has been a steep increase in sickness absence due to mental disorders since 2017, especially in the diagnostic groups of depression and anxiety disorders. While there has been a longer-term increase in sickness absence due to mental disorders in the age group 16–34 years, a steep increase has also been observed after 2016 among older age groups. These trends have been particularly alarming among women. However, as a similar trend was observed in all groups, the underlying causes must be related to factors that affect different gender and age groups rather similarly [2].

Several concurrent changes may drive the development, such as increasing demands at work and challenges of combining work with other schemes of life, less stigmatisation of mental health problems, changes in help-seeking behaviour and better recognition of mental health problems [6]. Also social comparison through social media may affect the trend [15]. On the other hand, due to a decreasing unemployment rate during 2016–2019, more people with health problems may have gained employment, and these problems may have been demonstrated through increasing sickness absence [16].

According to the Finnish Quality of Work Life Surveys, self-reported psychological symptoms such as tiredness, anxiety and stress, as well as time pressure and overall mental burden of work, have recently increased particularly among women and among younger workers [17]. Future studies need to investigate to what extent these developments explain the trend of rapidly increasing sickness absence in these groups, and what other societal and individual-level factors may play a part. The trend of increasing sickness absence due to mental disorders needs to be noted across all population groups as a sign of an emerging problem that may have long-lasting consequences on work ability and productivity. Also the current COVID-19 pandemic may entail mental health consequences such as depression and anxiety, caused by, for example, social isolation, uncertainty and work-related stress especially among healthcare workers [18, 19]. Thus, in the current situation, monitoring the development of sickness absence due to mental disorders in different population groups is ever more warranted.

## Supplemental Material

sj-pdf-1-sjp-10.1177_1403494821993705 – Supplemental material for Increase in sickness absence due to mental disorders in Finland: trends by gender, age and diagnostic group in 2005–2019 Click here for additional data file. Supplemental material, sj-pdf-1-sjp-10.1177_1403494821993705 for Increase in sickness absence due to mental disorders in Finland: trends by gender, age and diagnostic group in 2005–2019 by Jenni Blomgren and Riku Perhoniemi in Scandinavian Journal of Public Health
