# Supplementary material for: Increase in sickness absence due to mental disorders in Finland: trends by gender, age and diagnostic group in 2005–2019
Source: Scand J Public Health. 2021 Feb 20;50(3):318–22. doi: 10.1177/1403494821993705 (PMC9096587; doi:10.1177/1403494821993705)

**Supplemental Figure.** Age-standardized sickness absence rates due to major diagnostic groups in Finland, 2005–2019, in three age groups. Yearly sickness absence days per each insured person.

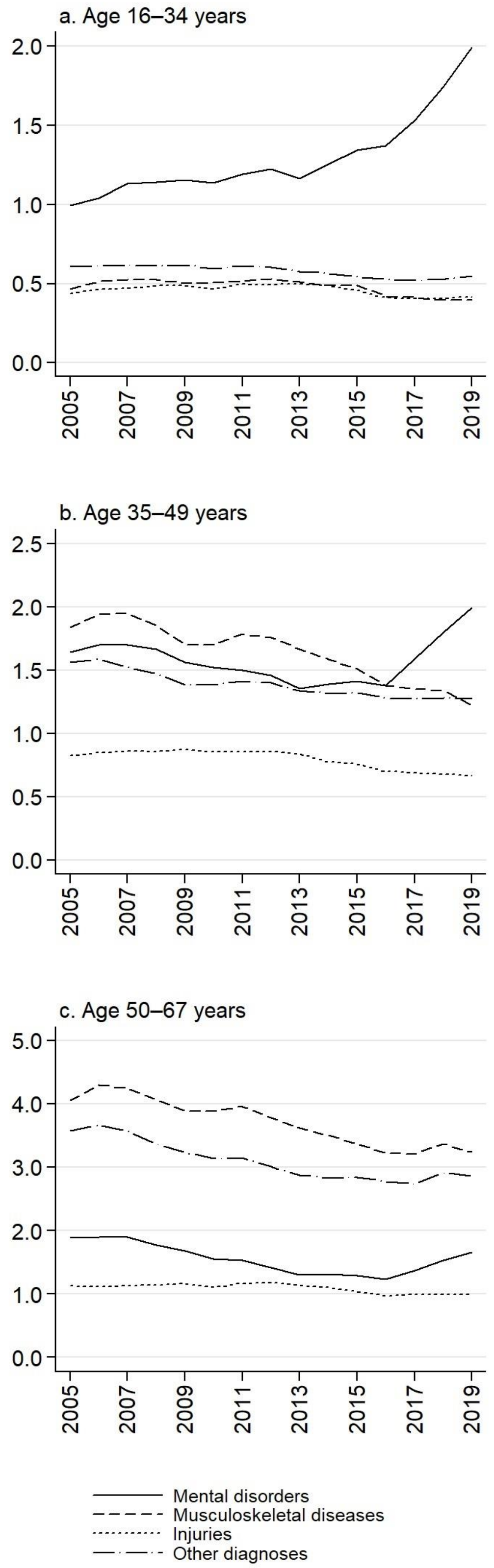

Supplement: sj-pdf-1-sjp-10.1177_1403494821993705 – Supplemental material for Increase in sickness absence due to mental disorders in Finland: trends by gender, age and diagnostic group in 2005–2019 [file sj-pdf-1-sjp-10.1177_1403494821993705.pdf]
